# Diseases of the Blood

**Published:** 1905-02-11

**Authors:** 


### Progress in Medicine and Surgery.

### DISEASES OF THE BLOOD.

(*Continued from page* 335.)

Leukæmia in Childhood.—The old division into splenic and lymphatic forms, based mainly on clinical differences, had to give way to a pathological classification of lymphatic and myelogenous forms founded on the chief type of cells found in the blood. More recent work still has shown that the differentiation of function between the bone marrow and adenoid tissue is not exclusive, and that a considerable amount of vicarious action can take place, so that the marrow no longer confines itself to the exclusive formation of granular cells, nor the adenoid tissue to the production of lymphocytes. This view applies with even greater force in childhood, where the specialisation of function is less stereotyped than in the adult. Myelogenous leukæmia is exceptionally rare in childhood, whilst the lymphatic form is comparatively frequent. Clinically the latter cases may be divided into three classes—those which exhibit profound anæmia with general glandular enlargement and a hæmorrhagic tendency in the later stages, those with a hæmorrhagic tendency from the first, and pseudo-scorbutic cases in which lesions in the buccal cavity are the most striking feature. The type of cell which predominates in the blood is the large lymphocyte, and pathologically there is overgrowth of adenoid tissue and replacement of the normal bone marrow by lymphoid tissue. No satisfactory explanation of the frequency of the lymphatic form, and the rarity of the myelogenous form, of leukæmia in childhood, has as yet been given. The former fact may be correlated, however, with the great extent and activity of the adenoid tissue in childhood. Mixed forms of leukæmia, intermediate in type between the lymphatic and the myelogenous types, are frequent in childhood; this is not surprising, since both types of cell probably arise from a common parent type of cell, which may give rise to either myelocytes or lymphocytes, whilst in the intermediate forms either the parent cells themselves enter the blood along with myelocytes, or produce both myelocytes and lymphocytes in excessive numbers. The trend of opinion is thus to classify leukæmia into myelocytic, lymphocytic, and mixed-cell forms rather than into myelogenous and lymphatic.

The Thymus is not possessed of any functions peculiar to itself and distinct from those of the histologically similar adenoid tissue met with throughout the body. It attains its infantile type by the end of the third month of intra-uterine life, and from then increases rapidly in functional activity, so that by the time of birth its weight, relative to that of the body, has reached its maximum. From then onwards it gradually undergoes a decline, though not ceasing to be functionally active until growth is complete. It may be taken as an index of the functional activity of the adenoid tissues, and of metabolism in general. Anything which interferes with nutrition reacts with special force on the thymus, *e.g.* in infantile marasmus it rapidly wastes. The association of sudden death with hypertrophy of the thymus has long been known, though unexplained.

Some have supposed it to be due to mechanical causes, others to a lymphatic dyscrasia, and a general feebleness of resistance.

Affections of the Lymphatic Glands may be divided into several varieties. There is first a multiple acute tubercular adenitis, which runs a comparatively short course marked by irregular pyrexia, often with periodic exacerbations; there is a more or less generalised enlargement of the lymphatic glands but not to any extent of the spleen, and a pronounced degree of anæmia, accompanied by more or less polynuclear leucocytosis. Secondly, Hodgkin's disease, which is characterised clinically by progressive enlargement of the lymph glands, and sometimes also of the spleen without any increase of the leucocytes. Pathological investigation of such cases shows that the distinction between cortex and medulla in the glands is abolished, the whole gland having a homogeneous appearance. The number of lymphocytes is much decreased and the framework of the gland has undergone hyperplasia with a conspicuous fibrillar reticulum, and its increase may amount to a true fibrosis. The endothelial cells in connection with the reticulum may attain a large size and may contain two or more large nuclei. All attempts to trace the presence of any one infective agent have failed, and it is possible that the disease is merely the reaction of adenoid tissue to any mild chronic irritant present in the circulation. Secondary infections, as by the tubercle bacillus may occur. The blood-picture is a variable one; though the total number of leucocytes is not increased there may be a relative increase of the polynuclears or of the lymphocytes. Information is wanted on the question whether the occurrence of a polynuclear leucocytosis is only observed in cases of secondary infections. Thirdly, lymphosarcoma may occur; it invades locally, spreads by the lymphatics, but not usually by the blood-vessels, and rarely forms true metastases. A marked lymphocytosis is often present. These three affections of the lymphatic glands are probably quite distinct pathological processes, and there is no relation between Hodgkin's disease and leukæmia. On the other hand, there is a certain resemblance between lymphosarcoma and the more chronic forms of leukæmia, and the disease termed chloroma is a connecting link between them. In chloroma there are multiple green lymphomata localised chiefly in the periosteum and neighbourhood of the bones, especially of the head, accompanied by a lymphoid transformation of the bone-marrow and a blood-picture resembling that of leukæmia. There are thus three processes—lymphosarcoma, chloroma, and leukæmia—closely allied and exhibiting a descending scale of malignancy.

